# Evaluation of collaborative TB/HIV activities in a general hospital in Addis Ababa, Ethiopia

**DOI:** 10.1186/1756-0500-5-67

**Published:** 2012-01-26

**Authors:** Aragie Kassa, Degu Jerene, Yibeltal Assefa, Azmera Teka, Abraham Aseffa, Amare Deribew

**Affiliations:** 1 Ministry of Health, Addis Ababa, Ethiopia; 2 HIV/AIDS prevention and control office, Addis Ababa, Ethiopia; 3 Private consultancy and research firm, Addis Ababa, Ethiopia; 4 Armauer Hansen Research Institute, Addis Ababa, Ethiopia; 5 Department of Epidemiology, Jimma University, Jimma, Ethiopia

## Abstract

**Background:**

Ethiopia has had mechanisms for TB/HIV collaborative activities since 2002. However, no published account has defined the role of these collaborative efforts in strengthening linkages between HIV and TB management units at the point-of-care level. Our objective was to assess the extent of linkages between the two programs at the patient management level at Zewditu Memorial Hospital in Addis Ababa, Ethiopia. Between January and December 2008, the registers of 241 TB patients were reviewed to determine the HIV testing rate, the treatment charts of 238 randomly selected patients were reviewed for providers' compliance with evaluation criteria, and exit interviews were conducted with 309 TB/HIV co-infected clients to validate providers' compliance.

**Results:**

From register review, it was determined that the HIV testing acceptance rate was 95%, and that 70% of patients received post-test counseling. A review of the patient chart revealed that of 51 patients with a complaint of cough, duration for cough was recorded in 35 (68.6%) cases and cough > 2 weeks was recorded in 25 (49.0%) cases. Seventy two percent (18 of 25) were linked for sputum microscopy. Linkage to cotrimoxazole prophylactic treatment was 81%, but only 47% of eligible patients were linked to isoniazid preventive therapy (IPT). Correct diagnosis was accomplished at a rate of 100% for smear positive pulmonary TB, 23% for smear negative pulmonary TB and 88% for extra pulmonary TB patients. Both chart review and exit interviews indicated that history of TB contact and cough > 2 weeks predicted TB disease.

**Conclusion:**

The rates of HIV testing and linkage to cotrimoxazole prophylactic therapy were high. Improvement is needed in the areas of recording patient information, screening HIV positives for TB, initiation of IPT, referral, linkages, and TB diagnostic capacity.

## Background

Tuberculosis (TB) is the leading killer of human immunodeficiency virus (HIV) infected individuals, and the overlapping epidemics have had a devastating impact on TB and HIV morbidity, mortality and control worldwide [1]. These TB and HIV co-epidemics require urgent and effective attention and demand a collaborative effort between TB and HIV programs employing different but complementary strategies. Both programs should be able to identify and manage both diseases [2,3].

However, the two programs are often separate at the level of patient care contributing to delayed diagnosis and linkage to care. TB and HIV programs must establish linkages to better utilize resources, avoid missed opportunities, and accelerate universal access to comprehensive TB and HIV prevention, treatment and care services [4].

In Ethiopia, 24% HIV sero prevalence is reported in TB patients from health facilities implementing TB/HIV services [5]. In 2000, of 51 consecutive cultures of proven TB patients in Addis Ababa, 47% were HIV Positive [6]. Nonetheless, no research in Ethiopia defined the collaborative activities since it started implementation in 2002. The objective of this study was to describe the level of linkage between TB and HIV programs in patients attending care and treatment in Zewditu Memorial Hospital in Addis Ababa.

## Methods

### Study setting

Zewditu Memorial Hospital (ZMH) is a general hospital located in Addis Ababa, Ethiopia. It has been providing antiretroviral therapy since 2003. Addis Ababa has a projected population of 3 million, and is divided in to 10 administrative sub cities and 99 localities [7,8].

As a standard of care, the Ethiopian national guidelines for TB/HIV collaboration advised offering HIV testing to those with cough > 2 weeks, provision of IPT to HIV positives screened negative for TB, cotrimoxazole prophylactic therapy (CPT) to co- infected patients, and regular TB screening of clients in HIV care [5].

### Study design and data collection

This cross-sectional study was conducted between January and December 2008. Three methods were used to evaluate the performance of the TB/HIV collaborative activities: review of registers of TB patients to determine the HIV testing rate, retrospective review of charts of patients co-infected with TB and HIV, and exit interviews. The registers of all the 241 TB patients who visited the clinic between January and December 2008 were reviewed, as well as the charts of 238 randomly selected TB/HIV co-infected patients aged 18 years and above and treated at the ZMH during the specified period. In addition, 309 randomly selected TB/HIV co-infected patients were interviewed during the data collection period.

Information was retrieved from TB and provider-initiated counseling and testing (PICT) registers and from HIV care/anti-retroviral treatment (ART) charts using a data abstraction checklist. Data from registers and charts included information on HIV testing and post test counseling of TB patients, referral to HIV care of HIV positive TB patients, screening of HIV positives for (cough, fever, and night sweating > 2 weeks), weight lost > 3 kg in the past 4 months, and history of TB contact in the past 1 year, sputum microscopy for acid-fast bacilli (AFB), sputum culture, lymph node fine needle aspiration (FNA) test, linkage to IPT and CPT. Structured questionnaire containing standard TB screening criteria(if the client had cough, fever, and night sweats for > 2 weeks, weight loss > 3 kg in the last 4 weeks, and TB contact in the past 1 year) were used for exit interview of clients how they screened for TB. The groups of patients included in the exit interview were those screened for TB in the past 1 year.

The data abstraction checklist and the exit interview questionnaire were adapted from the national TB/HIV guideline [5], and pretested before being applied for data collection. The questionnaire for exit interview was translated to Amharic (local language) and translated in English to maintain consistency. We used experienced and trained data collectors for all data collections activities.

### Evaluation criteria

The following indicators adapted from World Health Organization (WHO) guidelines were used to evaluate the level of linkage implemented between TB and HIV services: TB patients who are tested for HIV, HIV positive clients given IPT, co infected patients put on CPT, proportion of patients with cough > 2 weeks duration and sputum for AFB is ordered, proportions of TB suspect patients diagnosed correctly for TB [5,9].

TB screening of HIV positives included asking questions based on a combination of symptoms of the five TB screening criteria indicated in the national protocol. The screening checklist included cough, fever, night sweats (> 2 weeks), weight loss > 3 kg in the last 4 weeks, and history of TB contact in the past 1 year. If the client said yes for cough > 2 weeks or if no to cough > 2 weeks but yes to two or more of the other questions the patient was further evaluated [5].

Diagnosis of TB in HIV positive patients was based on the national TB/HIV guidelines. Pulmonary TB (PTB) diagnosis was established if at least one sputum smear-positive for AFB was detected. The algorithm for smear negative PTB diagnosis required at least 2 slides negative for sputum AFB, no response to broad spectrum antibiotic for 10-14 days, and radiographic abnormalities consistent with active TB; or, at least 2 slides negative for sputum AFB plus sputum culture positive for M. tuberculosis. Extra pulmonary TB (EPTB) diagnosis was based on FNA suggestive of, or consistent with, active extra pulmonary TB, one specimen from an extra pulmonary site culture positive for M.tuberculosis, or smear positive for AFB with the clinician's decision to treat the patient with a full course of anti-tuberculosis treatment.

### Statistical analysis

Quantitative data was sorted, cleaned, and coded after double data entry using the EPI-Info version 3.3.2 data entry program. Analysis was conducted with SPSS 16.0 (SPSS inc. Chicago, 2007). Quantitative data was analyzed by calculating percentage of indicators using descriptive statistics. Percentages were used for comparison. We used both bivariate and multivariate analysis to estimate demographic characteristics and TB screening criteria for specific outcome variables in the program. We determined the level of significance at *P *< 0.05.

### Ethical review

The study was approved by the ethics committee of Addis Ababa Regional Health Bureau. The purpose of exit interview was described and participation was fully voluntary and all study subjects gave verbal consent. Interview was done privately and data captured from various sources were kept confidentially.

## Results

### HIV testing among TB patients

The mean age of registered TB patients was 30.2 years [range 18-80 years; standard deviation (SD) 12.9]. Of 241 TB patients, 95% were tested for HIV. About 86% (68/79) of those tested positive received post-test counseling, 6% did not receive post-test counseling, and in about 8% the status of post-test counseling was missing (Table 1).

**Table 1:** Baseline characteristics of the study participants in Zewditu Memorial Hospital, Dec 2008

Characteristics		Number (%)
Gender		

	Female	125(51.9)

	Male	116(48.1)

Age		

	18-30	156(64.7)

	31-45	59(24.5)

	46-80	26(10.8)

TB patients offered HIV testing		

	Yes	230(95.4)

	No	11(4.6)

HIV test result		

	Positive	79(34.3)

	Negative	123(53.5)

	Not indicated	28(12.2)

Post test counseling for those tested for HIV		

	Yes	162(70.4)

	No	23(10.0)

	Not indicated	45(19.6)

Referred sero-positives for HIV care		

	Within facility	25(31.6)

	Other facility	30(38.0)

	Not indicated	24(30.4)

### Medical chart reviews for provider compliance

Mean age of patients whose charts were reviewed was 34.6 years (range 18-60; SD 8.1). With respect to educational attainment, 42.8% had completed primary school and 57.1% had completed secondary and above. About 46.2% were unemployed.

**Figure 1:**
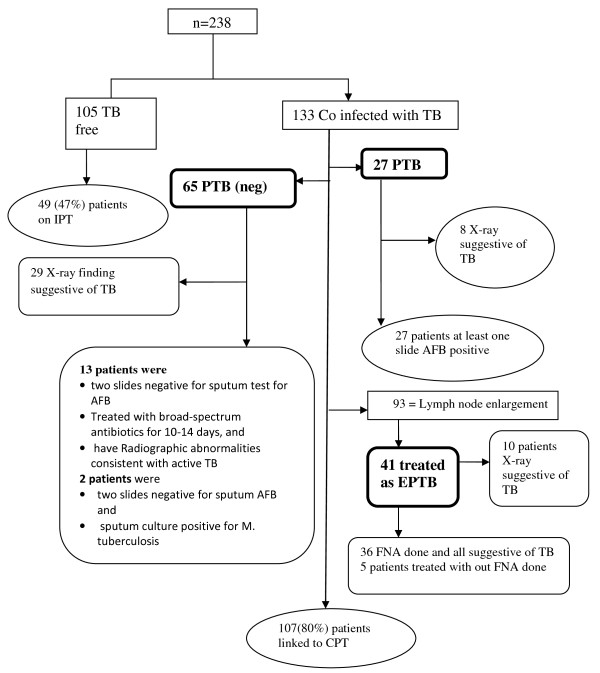
**Profile of tuberculosis diagnosis abstracted from chart review, Zewditu Memorial Hospital, Ethiopia (January-December, 2008)**. Out of 238 HIV positive patients charts reviewed, 133 were co-infected with TB. Of these 27 were sputum positive pulmonary TB (PTB), 65 were sputum negative pulmonary TB [PTB (neg)], and 41 were extra pulmonary TB (EPTB). The diagnostic algorithm indicated correct diagnosis were done for all PTB patients, 15 PTB (neg) patients and 36 EPTB patients. Among those diagnosed as PTB (neg), about 29 patients had X-ray done that was suggestive of TB. One hundred and five patients were ruled out for tuberculosis but linkage to IPT was done only for 49(47%) patients. Linkage to CPT of TB/HIV co-infected patients was 80%.

From 106 patients on ART and 132 patients on pre-ART category included in the study, 44(41.5%) and 89(67.4%) of patients were diagnosed for any form of TB respectively. Two hundred and four (85.7%) patients were asked for a history of cough. Fifty-one patients (25.0%) had complaint of cough and duration of cough was recorded for 35(68.6%). Cough lasting > 2 weeks was recorded for 25(49.0%) of patients and 18(72.0%) were linked for investigation of sputum for AFB. The rate of sputum AFB positive for cough lasting > 2 weeks was 29.6% with at least one slide positive for AFB. Of all the charts with diagnosis of TB, 27(11.3%) were diagnosed for PTB, 65 (27.3%) smear negative PTB, and 41(17.2%) were diagnosed for EPTB (Figure 1).

By chart review, one hundred and sixty three patients were symptom screen positive for fever and history of TB contact during the past year. Among these, 4(2.5%), 65 (39.9%), and 94(53.3), were not linked for radiographic examination, sputum test for AFB, and physical examination for further evaluation, respectively. For 171 patients who said "yes" for history of night sweating and TB contact at the same time, 4(2.3%), 70(40.9%), and 97(56.7%) were not linked to radiographic examination, sputum test for AFB, and physical examination for additional evaluation of tuberculosis, respectively. About 14.3% of patients included in the chart review were not screened for history of TB contact in the past year.

Correct diagnosis was done at the rate of 100% for smear positive PTB, 23% for smear negative PTB and 87.8% for EPTB patients. In multivariate analysis history of TB contact in the past 1 year (96.3% for smear positive PTB, 90.8% for smear negative PTB and 92.7% for EPTB) and cough > 2 weeks were predictors of TB disease (Table 2).

**Table 2:** Potential predictors of TB infection for chart review, Zewditu Memorial Hospital, Dec 2008.

Predictor variable	Category	Crude OR	*P*-value*	Adjusted OR	*P*-value
Age	18-34	Referent	0.31	Referent	
			
	≥ 35	1.27 (0.76-2.11)		1.22 (0.71-2.07)	0.47

Occupation	Unemployed	Referent		Referent	
	
	Employed	0.96 (0.74-1.26)	0.86	0.82 (0.48-1.42)	0.32

Education	No education	Referent		Referent	
	
	Primary & above	0.77 (0.48-1.31)	0.26	1.62 (0.63-4.17)	0.32

History of TB contact	No	Referent		Referent	
	
	Yes	3.06 (1.36-6.89)	0.007	2.81 (1.22-6.45)	0.02

Cough > 2 weeks	No	Referent		Referent	
	
	Yes	3.79 (1.46-9.86)	0.006	3.77 (1.44-9.87)	0.01

*Level of significance at *p *< 0.05

### Findings from the exit interview

A total of 309 respondents participated in the exit interview. The mean age of the participants was 34 years (range 18-55; SD = 6.4); 77.7% were aged < 39 years; and 57% were females. 63.8% of respondents were secondary and above level of education and 30.4% of them were unemployed. Over a half (52.1%) of the respondents were co-infected with TB in the past 3 years after they knew their HIV sero status of which 103(64.0%) were taking CPT. Out of 162 co-infected patients 99 (61%) have a history of TB contact in the past 1 year. From the multivariate analysis, history of TB contact in the past year was a major predictor of TB disease (Table 3). About 46.6% of interview participants were not screened for history of TB contact in the past year.

**Table 3:** Analysis of TB contact as potential predictor to TB disease for exit interview of PLHIV, Zewditu Memorial Hospital, Dec 2008.

Predictor variable	Category	Crude OR	*P*-value*	Adjusted OR	*P*-value
TB contact	No	Referent		Referent	
	
	Yes	1.93 (1.23-3.03)	0.004	2.76 (1.22-6.39)	0.006

Age	18-34	Referent		Referent	
	
	≥ 35	0.94 (0.42-1.55)	0.81	1.04 (0.63-1.71)	0.87

Occupation	Unemployed	Referent		Referent	
	
	Employed	0.89 (0.46-1.72)	0.61	1.095 (0.68-1.75)	0.71

Education	No education	Referent		Referent	
	
	Primary & above	0.76 (0.35-1.66)	0.492	1.13 (0.50-2.51)	0.77

* Level of significance at *p *< 0.05

## Discussion

This study uncovered high rates of HIV testing and linkage to CPT among TB patients. This can likely be attributed to the scaled up of PICT implementation in the study setting and show commendable progress [10]. However, the low IPT uptake, incompleteness of TB and PICT registers, and inadequate TB diagnostic capacity need more attention.

Although cough and history of TB contact in the past year were found to be a major predictors for symptom screening of tuberculosis in chart review (Table 2) and in exit interviews (Table 3) respectively, both predictors were not consistently used as TB screening criteria. Poor adherence to the national guidelines in treating smear negative PTB was discovered, with less than one quarter of patients being diagnosed correctly. The same was true for EPTB patients.

The World Health Organization (WHO) recommends routine offering of HIV testing and CPT to all HIV patients co-infected with TB. CPT is recommended to all HIV positives co-infected with TB [3]. In a setting with significant challenges HIV testing and CPT rates of 95 and 81% respectively concurs with these global recommendations. Also, the HIV testing rate supersedes that reported by neighboring Kenya [11]. The CPT Uptake in our study setting exceeds that documented in Uganda [12].

In concurrence with global reports (WHO 2010 report), the IPT uptake rate was low [13]. Although the guidelines recommend IPT as part of the standard care for HIV patients without active TB, low uptake continues to be challenge. This is not unique to Ethiopia. Fear of isoniazid (INH) resistance, failure to exclude active TB, poor adherence, shortage of supplies, and provider attitudes appear to impede its scaled up implementation [14]. This highlights the need to continue to advocate for improved delivery of IPT. More simplified guidelines are now available to guide its strengthened implementation [15]. Additional observed weaknesses of the program such as failure to utilize symptom screening, poor adherence to national guidelines with regard to treating smear negative PTB and EPTB can be addressed through continuous education and mentoring of health care providers.

Culture for Mycobacterium tuberculosis increases the likelihood of diagnosis [16]. Nonetheless, due to costs of sputum culture and FNA the proportion of patients benefited from both tests was very low. About 56.5% people living with HIV included in the study were unemployed and hence unable to make payments for the indicated laboratory investigations. A recent prospective study evaluating the prevalence of active TB in HIV infected patients with a CD4 cell count of > 200 cells/μl reported that 29% of those with culture- confirmed pulmonary TB had normal radiography and clinical examination [17].

It appears that payments related with sputum culture and FNA impede compliance with the national protocol and the TB/HIV linkage activities in the study setting. Exempting patients from payments associated with these tests can create conditions conducive to resolving existing diagnostic problems. There is a need to strengthen laboratories with better TB diagnostic facilities, primarily for performing sputum culture and fluorescence microscopy. This could be done by assisting the existing laboratories in increasing the volume of testing and also providing financial support for them to conduct targeted tests such as sputum culture and FNA free of charge. Adding sputum culture to the package of care for people living with HIV (PLHIV) will substantially increase case detection, which is part of Ethiopia's commitment to improve TB and HIV care.

## Conclusion

The high rates of HIV testing and linkages to CPT are encouraging indicators of progress in the implementation of TB/HIV collaborative activities in the study setting. However, low rates of IPT, poor diagnostic capacity, and inadequate documentation at TB clinics need to be addressed. Structural barriers to TB diagnostic facilities such as fees for TB culture need to be solved, and simpler diagnostic techniques should be made available in such settings. Periodic evaluation of the progress using larger sample sizes among nationally representative patient populations would contribute to improved quality of care to TB/HIV co-infected patients in Ethiopia.

## Authors' contributions

AK was involved in the conception, design of the study, coordination of data collection, analysis, and drafting of the manuscript. AT was involved in design, field work, and review of the article. DJ, AD and YA participated in the design and review of the article. AA participated in the review of the article. All authors read and approved the final manuscript.

## Competing interests

The authors declare that they have no competing interests.
